# Characterizing the Crosstalk of NCAPG with Tumor Microenvironment and Tumor Stemness in Stomach Adenocarcinoma

**DOI:** 10.1155/2022/1888358

**Published:** 2022-10-03

**Authors:** Zheng Xiang, Genlan Cha, Yihao Wang, Jikai Gao, Jianguang Jia

**Affiliations:** ^1^Department of Oncology, The First Affiliated Hospital of Bengbu Medical College, Bengbu, China 233000; ^2^Department of Radiotherapy, The First Affiliated Hospital of Bengbu Medical College, Bengbu, China 233000

## Abstract

**Background:**

Nonstructural maintenance of non-SMC condensin I complex subunit G (NCAPG) exerts critical effects on cancer progression. However, its biological roles in tumorigenesis and metastasis remain unclear. Thus, we aimed to assess the prognostic utility of NCAPG in stomach adenocarcinoma (STAD) and its potential as a tumor biomarker.

**Methods:**

Pan-cancer expression profile dataset from public databases and corresponding clinical information were extracted. Single-sample gene set enrichment analysis (ssGSEA) was performed for the evaluation of immune correlations pan-cancer. Subsequently, we focused on STAD and evaluated the methylation profiles, copy number variants (CNVs), and single nucleotide variants (SNVs). Immune features were analyzed between high and low NCAPG expression groups. Differential analysis was performed between high and low expression groups to identify differentially expressed genes (DEGs). Prognostic DEGs were screened by univariate analysis, and an NCAPG-based risk model was constructed based on the prognostic DEGs and LASSO analysis.

**Results:**

NCAPG expression in STAD was significantly and positively correlated with four immune checkpoints, namely, CTLA4, PDCD1, LAG3, and CD276, but was negatively correlated with the infiltration of most immune cells. High and low NCAPG expression groups had differential overall survival, tumor mutation burden, and differential enrichment of therapeutic-related pathways. An immune risk scoring model related to NCAPG expression and immune score was constructed which showed a favorable performance in predicting STAD prognosis as well as predicting the response to immunotherapy. In addition, we found a higher mRNA stemness index (mRNAsi) in the high-risk group and a positive correlation between NCAPG expression and mRNAsi.

**Conclusion:**

NCAPG was suggested to be involved in the regulation of tumor microenvironment in STAD. High NCAPG expression was related to high tumor stemness and good prognosis. The immune risk model had a potential to predict STAD prognosis and help directing therapeutic treatment.

## 1. Introduction

Cancer is a major reason for the deterioration of quality of life globally and is the top cause of mortality; to date, there is no cure for cancer [1]. Immunotherapy has emerged as a prominent treatment option for cancer in recent years; in particular, immune checkpoint blockade agents are promising [2]. Using improved and updated datasets available through public databases such as The Cancer Genome Atlas (TCGA), new immunotherapeutic targets can be identified by performing pan-cancer gene expression analysis and evaluating their correlation with relevant signaling pathways and clinical prognosis [3].

The possible regulatory role of cohesin complexes (condensin complexes) in several cancer types including colon adenocarcinoma and hepatocellular carcinoma has been recently described. In cancer, these condensin complexes exert various impacts on the progression and drug resistance [4]. Nonstructural maintenance of non-SMC condensin I complex subunit G (NCAPG) is essential for chromatin segregation [5]. NCAPG is encoded by the NY-MEL-3 gene and is mapped to chromosome 4p 15.32 in humans [6]. In prostate cancer, a high NCAPG expression is related to a poor prognosis [7], and its knockdown combined with temozolomide treatment results in coinhibitory impacts on advanced pediatric glioma cells [8]. NCAPG is a hub protein, showing high connectivity in the protein-protein interaction (PPI) network in hepatocellular carcinoma and promotes cellular proliferation [9, 10]. Additionally, studies on stomach adenocarcinoma (STAD) suggest that through the Wnt/*β*-catenin signaling pathway by promoting epithelial-mesenchymal transition, NCAPG overexpression inhibits apoptosis in cardia adenocarcinoma [11]. Moreover, dysregulation of KNL1, miR-1179, NCAPG, miR-193b-3p, and miR-148a-3p may lead to STAD progression [12]. However, the mechanism underlying NCAPG-mediated proliferation in these cancers remains unknown, and its utility as a tumor-associated biomarker warrants further investigation.

The potential impact of NCAPG on several cancer types warrants clarification. We used two databases, Gene Expression Omnibus (GEO) and TCGA, to assess NCAPG expression in different types of cancers and their relationship with prognosis. We also investigated the immune correlation with NCAPG expression for 33 cancer types and subsequently found the strongest relationship for STAD. Next, we analyzed the potential associations between NCAPG and mutational types, DNA methylation profile, tumor mutation burden (TMB), immune infiltration levels, and clinical responses. We performed a PPI analysis of immune-associated differential genes with NCAPG to investigate its biological functions in the tumor. Finally, an immune risk score (IRS) model was constructed and the performance of the related score was better than that of the TIDE score. In conclusion, our results suggested that NCAPG may underlie implications as a prognostic factor and a potential biomarker for STAD. Thus, we provide insight into the role of NCAPG in STAD immunotherapy.

## 2. Methods

### 2.1. Data Source and Preprocessing

The bioinformatics analysis in this study was partially supported by Sangerbox (http://vip.sangerbox.com/) [13]. Thirty-three TCGA pan-cancer datasets comprising RNA sequencing (RNA-seq) expression profiles, survival information, and somatic mutational profile were extracted from https://xenabrowser.net/ (UCSC Xena). The RNA-seq data in fragments per kilobase million (FPKM) were converted to the transcripts per million (TPM) format, following which log_2_ transformations were performed. The downloaded somatic mutation data were already processed by mutect analysis. Finally, following the processing of the copy number variation (CNV) data by the GISTIC algorithm, these were extracted from UCSC Xena, while the methylation profiles were obtained from the LinkedOmics data portal (http://linkedomics.org).

We obtained the STAD-GEO cohort from https://www.ncbi.nlm.nih.gov/geo/ (GEO database), i.e., GSE66229, GSE84437, GSE26942, GSE13861, GSE28541, and GSE26253 comprising detailed survival data. The STAD sample data were preserved.

Three immunotherapy-associated cohorts, namely, GSE135222 (NSCLC), GSE91061 (melanoma), and GSE78220 (melanoma), were extracted. Complete expression data and corresponding detailed clinical information for a STAD immunotherapy-associated cohort (IMvigor210) were obtained using the Creative Commons 3.0 license (from http://research-pub.Gene.com/imvigor210corebiologies/).

### 2.2. Tumor Immune Microenvironment and Immune Infiltration Analysis

The single-sample gene set enrichment analysis (ssGSEA) in GSVA R package [14] was used to deal with the problem that single sample cannot perform GSEA. Therefore, the extent of multiple immune cell infiltration was estimated with the ssGSEA function in the GSVA package in R software. Using the Estimation of STromal and Immune cells in MAlignant Tumor tissues using the Expression data method (ESTIMATE), the tumor immune microenvironment score of samples was assessed [15]. Stromal, Immune, and ESTIMATE scores were computed and the differential distributions were compared.

### 2.3. Functional Enrichment Analysis

The limma package was utilized for differential expression analysis [16], and differentially expressed genes (DEGs) were identified based on false discovery rate (FDR) < 0.05 with |log_2_(fold change)| > 1.

Gene set containing the GSEA input file comprising the expression profile data was obtained (c2.cp.kegg.v7.0.symbols.gmt). Enriched pathways were selected, and GO annotation for DEGs was performed using the WebGestaltR package in R (v0.4.2) with *P* < 0.05 considered statistically significant [17].

### 2.4. Construction of the PPI Network

STRING (web version v11.0; https://string-db.org/) database was queried for the known and predicted PPIs. It is a database comprising information from 2031 species and 9.6 million proteins with 13.8 million interactions. The PPI network was constructed to uncover the core regulatory genes and visualized by using Cytoscape.

### 2.5. Construction of the IRS Model

In the GSE66229 dataset, random sampling was performed with a sampling ratio of train : test = 1 : 1. Univariate analysis was performed in the training dataset, and prognostic genes were obtained (*P* < 0.05). The best genes were further selected using LASSO. The R package, glmnet [18], was utilized to build the LASSO regression model with prognostic genes as the input.

Based on the formula defined by the sample risk score, the risk-related prognostic risk score (RiskScore) was computed for each sample as follows: RiskScore = *Σβ*_*i*_ × Exp_*i*_, where Exp_*i*_ indicates the gene signature expression and *β* is the corresponding gene LASSO regression coefficient. According to the median, patients were classified into high and low RiskScore groups. Survival curves were plotted for prognostic analysis by the Kaplan-Meier method, and the significance of differences was determined using a log-rank test. ROC analysis of RiskScore was performed using the timeROC package [19] in R for prognostic classification.

### 2.6. TIDE Analysis for the Effectiveness of Immunotherapy

Tumor Immune Dysfunction and Exclusion (TIDE) [20] analysis is utilized to identify biomarkers to predict the therapeutic responses to immune checkpoint inhibitors/drugs by a comprehensive analysis of several tumor expression profiles (http://tide.dfci.harvard.edu/). The sensitivity to immune checkpoint inhibitors is obtained as a TIDE score.

### 2.7. Calculation of Tumor Stemness

The expression data of embryonic stem cells (ESCs) and induced pluripotent stem cells (iPSCs) was downloaded from the Progenitor Cell Biology Consortium (PCBC) database (https://www.synapse.org/pcbc) [21]. The Ensembl IDs of SC samples were transferred to gene symbols. Only the protein genes were remained. A total of 78 SC samples were included. Gelnet (v1.2.1) R package (https://cran.r-project.org/web/packages/gelnet/index.html) was applied to analyze the mRNA stemness index (mRNAsi) of SCs. For the expression data, mean centering was used to normalize the samples. One-class logistic regression (OCLR) was conducted to calculate the weight vector of each gene. Then, we predicted the mRNAsi of TCGA samples based on the Spearman correlation analysis between the expression data and the weight vector of genes. The Spearman correlation coefficients (coef) were normalized according to the formula mRNAsi = [coef − min(coef)/max(coef)], where min(coef) represents the minimum coefficient and max(coef) represents the maximum coefficient. Finally, mRNAsi ranging from 0 to 1 was obtained.

## 3. Results

### 3.1. Pan-Cancer Immunological Correlation with NCAPG

**Figure 1:**
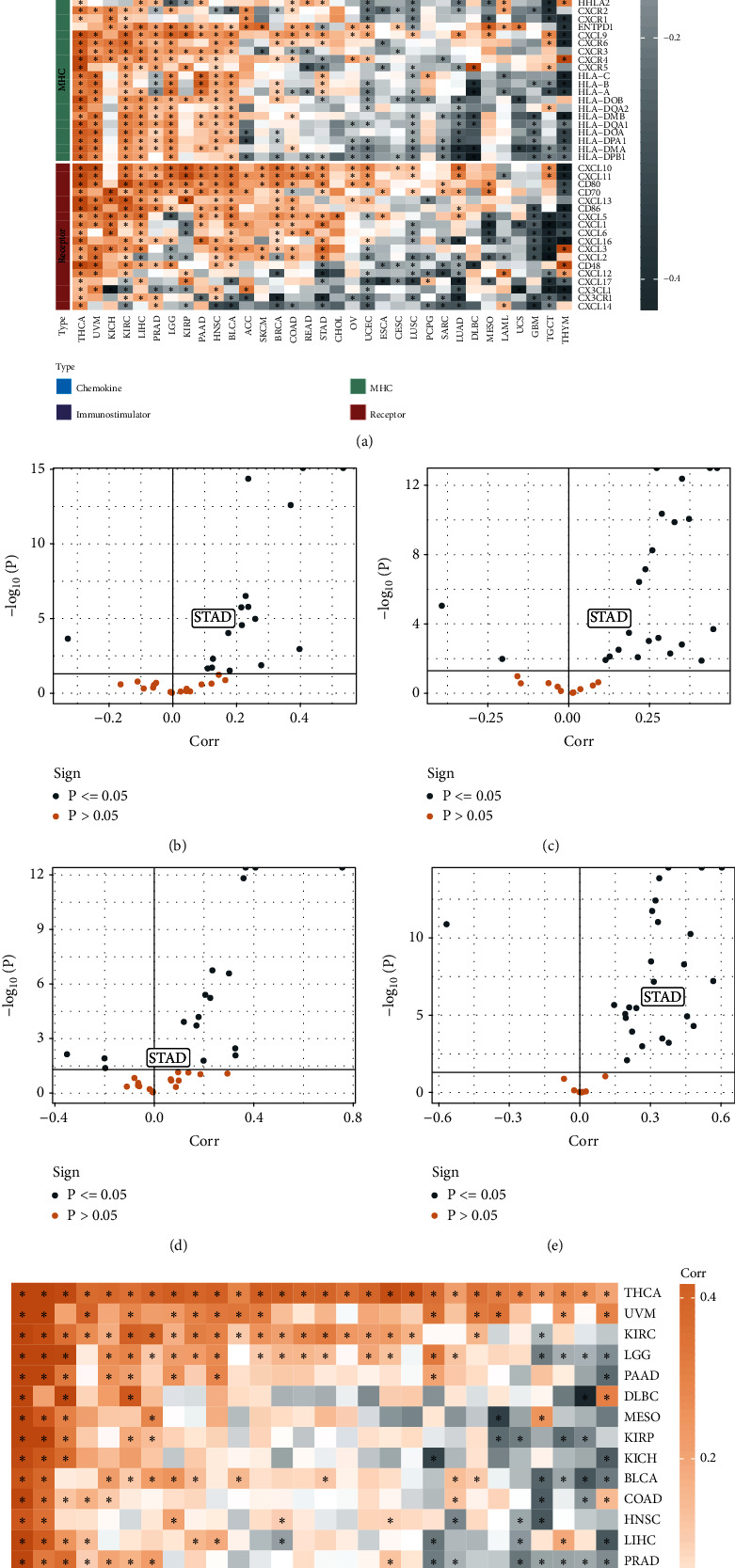
Pan-cancer immunological correlation with gene NCAPG. (a) Correlation between NCAPG and immunomodulators (chemokines, receptors, MHC, and immunostimulants). (b–e) Correlation between NCAPG and four immune checkpoints, namely, PDCD1, CTLA4, CD274, and LAG3. Points represent the cancer types. The *y*-axis represents the Pearson correlation coefficient, while the *x*-axis represents -log_10_ (*P* value). (f) Spearman correlation coefficients between NCAPG gene expression and 28 tumor-associated immune cells in 33 different cancer types are represented by colors. Asterisks indicate statistically significant *P* values for Spearman correlation analysis (^∗^ represents *P* < 0.05).

**Figure 2:**
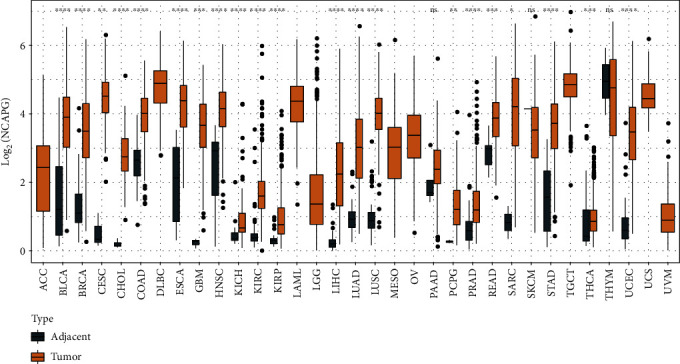
Pan-cancer differential expression of gene NCAPG between cancer and para-cancer tissues. Yellow represents significantly high expression in tumors (^∗^, *P* < 0.05; ^∗∗^, *P* < 0.01; ^∗∗∗^, *P* < 0.001; ^∗∗∗∗^, *P* < 0.0001; ns, *P* > 0.05).

Four types of immunological characteristics, namely, chemokines, immunostimulators, MHCs, and receptors, were identified from the literature [22]. The Spearman correlation coefficient was computed between NCAPG and the genes of immunological characteristics in pan-cancer data, and the correlations varied significantly across different cancer types, such as UVM (uveal melanoma), KICH (kidney chromophobe), and THCA (thyroid carcinoma) tumors showing positive correlation, while THYM (thymoma), TGCT (testicular germ cell tumors), and others exhibiting negative correlation (Figure 1(a)). We evaluated the correlation of four key immune checkpoint genes, namely, PDCD1, CTLA4, CD276, and LAG3, with NCAPG in different tumors by Pearson correlation analysis. The result exhibited that they were all positively correlated with NCAPG expression in STAD (Figures 1(b)–1(e)). We evaluated 28 immune cell scores in different cancer types by ssGSEA and calculated their correlation with NCAPG. NCAPG gene expression in STAD showed a significant negative correlation with the immune scores of 16 immune cells (Figure 1(f)). We assessed the gene expression of NCAPG in pan-cancer and found significant differences of NCAPG expression between tumor samples and adjacent samples (Figure 2). NCAPG expression was elevated in 21 tumor samples, including HNSC (head and neck squamous cell carcinoma), CHOL (cholangiocarcinoma, bile duct cancer), CESC (cervical squamous cell carcinoma and endocervical adenocarcinoma), BLCA (bladder carcinoma), ESCA (esophageal carcinoma, esophageal cancer), COAD (colon adenocarcinoma, colon cancer), BRCA (breast invasive carcinoma), KICH (kidney renal clear cell carcinoma), GBM (glioblastoma multiforme), LIHC (liver hepatocellular carcinoma), KIRC (kidney renal clear cell carcinoma), KIRP (kidney renal papillary cell carcinoma), LUSC (lung squamous cell carcinoma), LUAD (lung adenocarcinoma), PRAD (prostate adenocarcinoma), PCPG (pheochromocytoma and paraganglioma), SARC (sarcoma), UCEC (uterine corpus endometrial carcinoma), READ (rectum adenocarcinoma, rectal adenocarcinoma), STAD, and THCA.

According to the results of pan-cancer analysis, NCAPG correlated with the four types of immunological signatures as well as four important immune checkpoints in STAD. The expression of NCAPG was significantly lower in para-cancer relative to STAD samples. Furthermore, survival time between low and high expression groups showed significant differences in most of the tumors (Fig. S1). Therefore, we were interested in elucidating the role of NCAPG in STAD.

### 3.2. Analysis of SNVs, CNVs, and Methylation Profile in STAD

**Figure 3:**
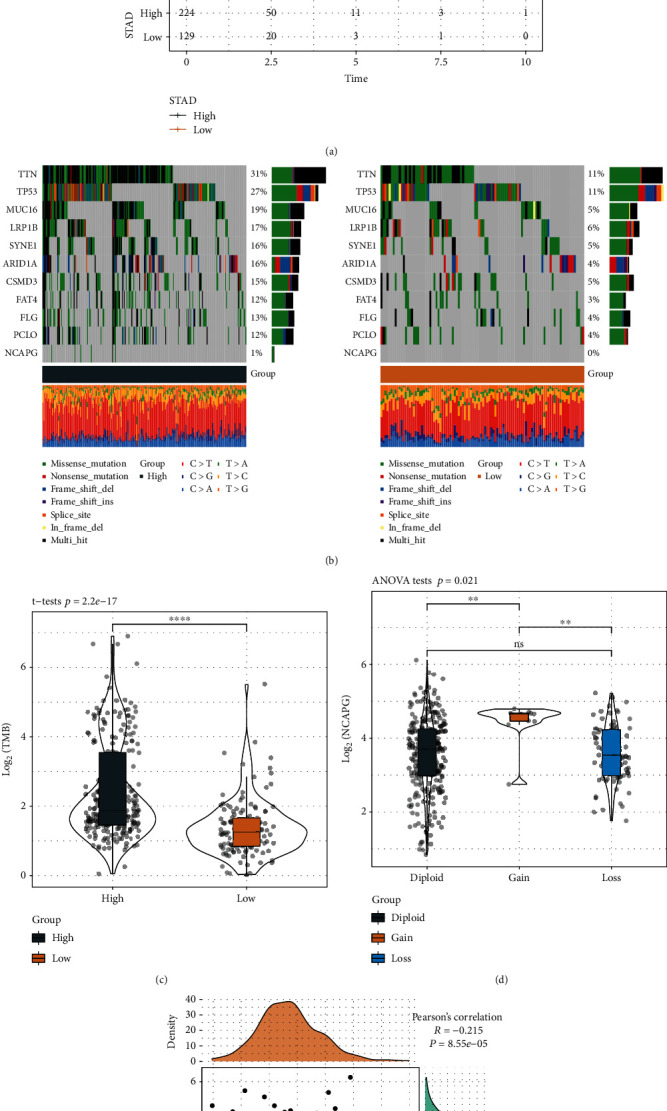
Analysis of SNVs, CNVs, and methylation profiles in STAD. (a) KM curve with 0 as the cutoff after expression *z*-score transformation; (b) mutational distribution for the top 10 genes with the highest mutation frequency in NCAPG expression subgroups; (c) comparison of TMB distribution between NCAPG expression subgroups; (d) NCAPG amplification subgroups based on expression differences; (e) correlation analysis for NCAPG expression and methylation status (^∗^, *P* < 0.05; ^∗∗^, *P* < 0.01; ^∗∗∗^, *P* < 0.001; ^∗∗∗∗^, *P* < 0.0001; ns, *P* > 0.05).

The STAD samples were classified into two groups with the best cutoff for NCAPG expression determined by survminer R package (Figure 3(a)). The high NCAPG expression group showed a more favorable prognosis, indicating that NCAPG may be a protective factor for STAD. Next, we plotted the 10 genes with the highest mutation frequencies between the expression groups. The mutation frequencies of TP53, TTN, SYNB1, RB1, KMT2D, and ARID1A were higher in the low expression group (Figure 3(b)). We then assessed the differences in the TMB of NCAPG between the groups. TMB was markedly higher in the NCAPG high expression group relative to the NCAPG low expression group (Figure 3(c)).

NCAPG expression in the presence of CNV amplifications versus deletions was significantly different from that in the case of the normal copy number, i.e., significantly upregulated expression was associated with CNV amplification while significantly downregulated expression was associated with CNV deficiency (*P* < 0.0001, Figure 3(d)). Meanwhile, the expression of NCAPG correlated negatively with the methylation profile of NCAPG (Figure 3(e), *R* = −0.215).

### 3.3. Comparative Analysis of the Immune Profiles between NCAPG Expression Groups in STAD

**Figure 4:**
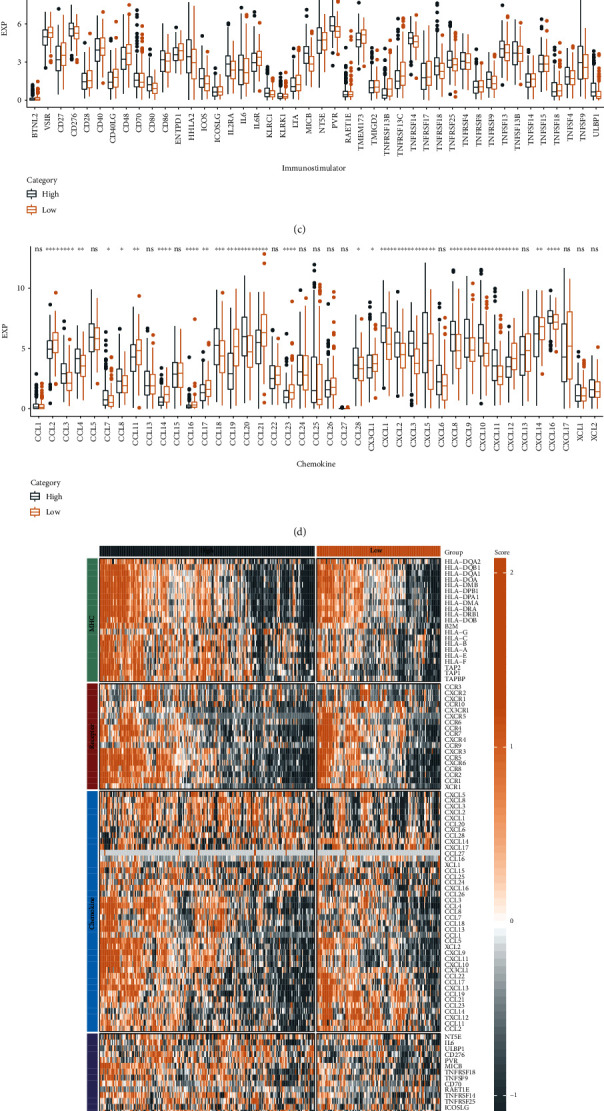
Comparative analysis for immune profiles of NCAPG subgroups in STAD. (a–d) Expression differences of different types of genes (chemokines, receptors, MHC, and immunostimulants) between NCAPG subgroups in STAD; (e) heat map of differential expression among gene types (chemokines, receptors, MHC, and immunostimulants) in STAD (^∗^, *P* < 0.05; ^∗∗^, *P* < 0.01; ^∗∗∗^, *P* < 0.001; ^∗∗∗∗^, *P* < 0.0001; ns, *P* > 0.05).

We analyzed the differential expression of four types of genes including chemokines, immunostimulators, MHCs, and receptors, between the two NCAPG expression groups. Among the receptors, genes including CCR7, CCR10, and CXCR5 were significantly higher in the NCAPG low expression group relative to the NCAPG high expression group (Figure 4(a)). In contrast, among the MHCs, genes including HLA-A, HLA-B, and HLA-C were significantly lower in the NCAPG low expression group relative to the NCAPG high expression group (Figure 4(b)). Among the immunostimulators, genes including CD276, CD80, and ICOS were significantly lower in the NCAPG low expression group relative to the NCAPG high expression group (Figure 4(c)). Finally, among the chemokines, CCL3, CCL4, and CCL18 were significantly higher in the NCAPG high expression group than in the low expression group (Figure 4(d)). Overall, the four key immune checkpoint genes differed significantly between the two evaluated groups (Figure 4(e)).

**Figure 5:**
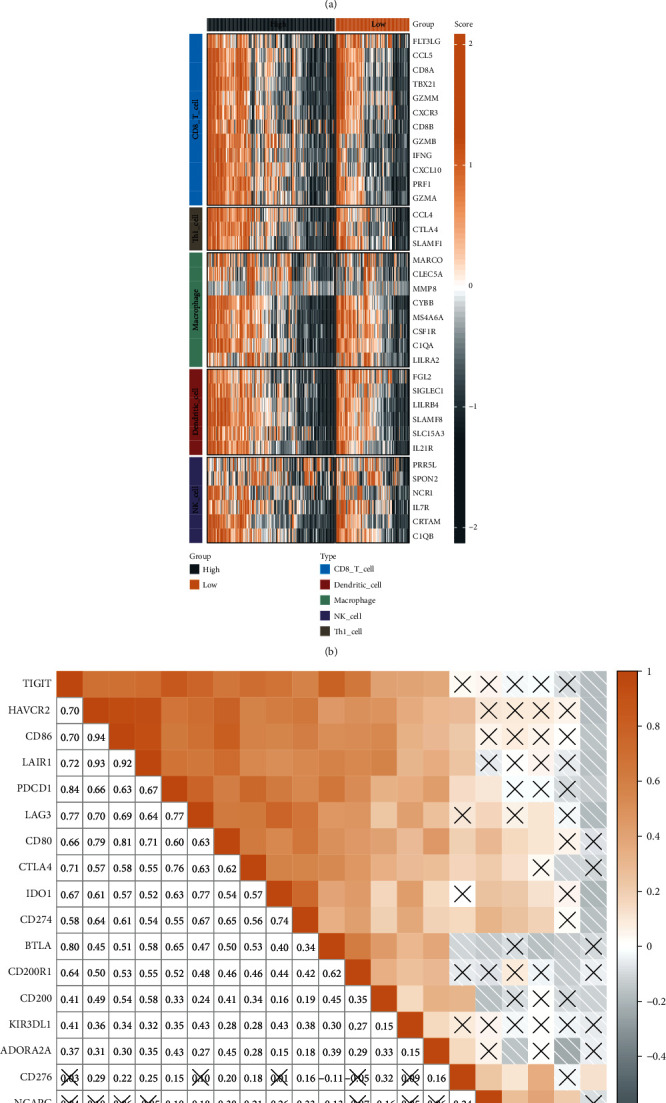
Differences in the distribution of immune cell scores between NCAPG subgroups. (a) Differences in immune cell scores between the high and low NCAPG groups. (b) Differences in five effector genes among five TIICs (CD8+ T cells, NK cells, macrophages, Th1 cells, and dendritic cells) between the high and low NCAPG groups. (c) Correlation between NCAPG and immune checkpoints. Colors and values indicate Spearman correlation coefficients (X represents *P* > 0.05) (^∗^, *P* < 0.05; ^∗∗^, *P* < 0.01; ^∗∗∗^, *P* < 0.001; ^∗∗∗∗^, *P* < 0.0001; ns, *P* > 0.05).

We compared the distributional differences of 28 immune cell scores between the NCAPG group, and 16 of them showed significant differences with high scores in the NCAPG low expression group (Figure 5(a)). We then performed immune infiltration analysis in STAD, calculated the association between immune infiltration scores and NCAPG expression, and performed a differential analysis of marker genes in five cell types, namely, dendritic_cell, CD8_T_cell, Th1_cell, NK_cell, and macrophage. In the NCAPG low expression group, FLT3LG was markedly upregulated (Figure 5(b)). We also assessed the association between immune checkpoints and NCAPG expression; significant slight positive correlations with immune checkpoints including CD80, CTLA4, IDO1, and CD274 were recorded (Figure 5(c)).

### 3.4. NCAPG Expression Predicts the Clinical Responses to Immune Checkpoint Blockade (ICB) in STAD

**Figure 6:**
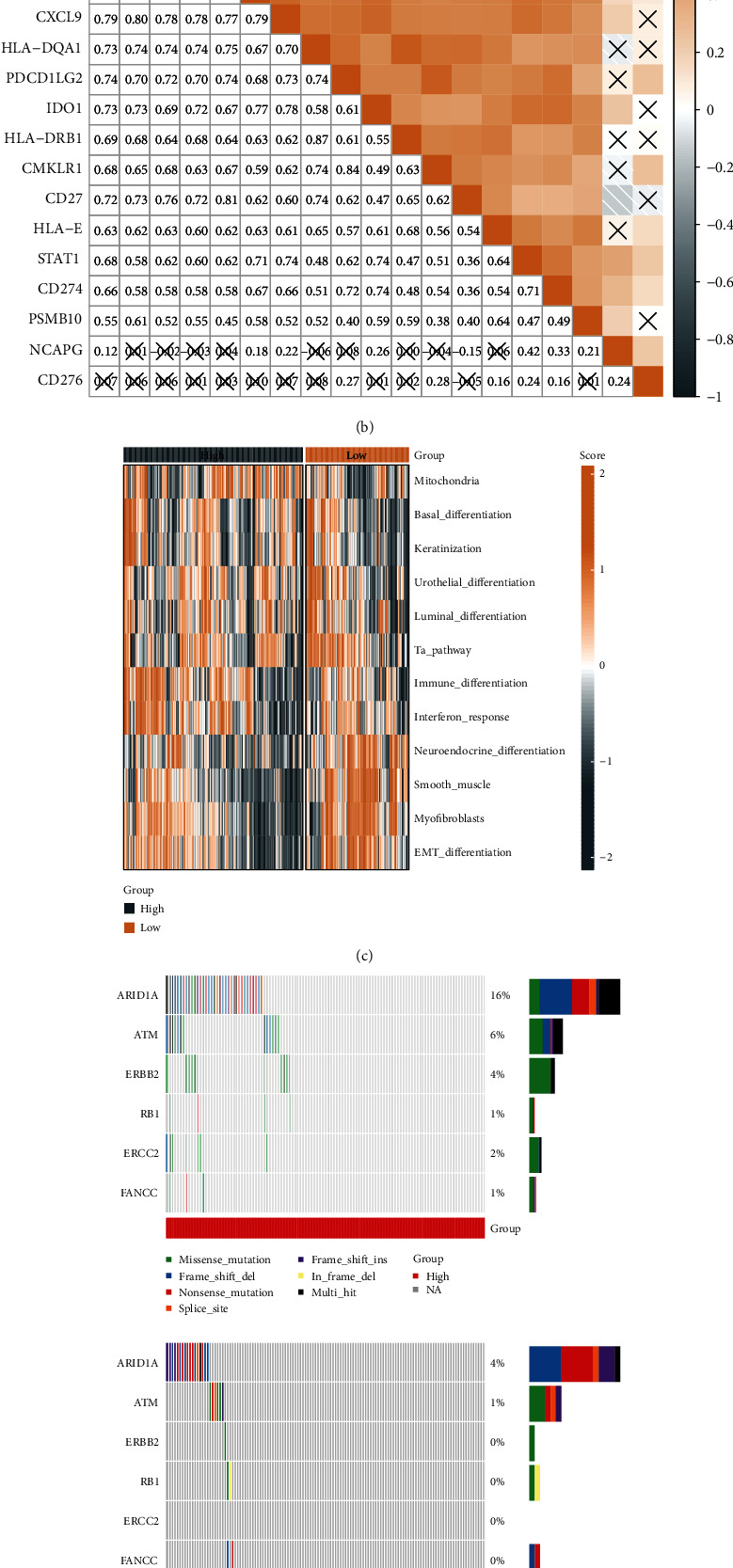
NCAPG immune signature analysis. (a) Correlation between NCAPG and pan-cancer T cell inflammation scores. (b) Pearson correlation analysis between NCAPG and immune checkpoints (X represents *P* > 0.05). (c) Correlation between NCAPG and molecular subtypes and features of STAD using seven different algorithms. (d) Mutational profiles of genes associated with neoadjuvant chemotherapy between low and high NCAPG groups. (e) Correlation between NCAPG and enrichment scores for several treatment features including targeted and radiation therapies (^∗^, *P* < 0.05; ^∗∗^, *P* < 0.01; ^∗∗∗^, *P* < 0.001; ^∗∗∗∗^, *P* < 0.0001).

The correlation between NCAPG expression and pan-cancer T cell inflammation score was assessed; but no significant correlation was observed (Figure 6(a)). Additionally, in subgroups with different ICB responses, the correlation between NCAPG expression and immune features (immune checkpoints, immunotherapy-related features, and expression of TIIC effector genes and immunomodulators) (Figure 6(b)) was assessed; NCAPG expression was not correlated with any of these features.

The association of scores of STAD tumors with immune-related pathways was evaluated; between the two NCAPG expression groups, significant differences in the associated immune pathways were noted (Figure 6(c)). For instance, smooth muscle and myofibroblasts pathways showed a predominant negative association with high NCAPG expression, while the trend was opposite in the low NCAPG group. We compared the mutation frequencies in ARID1A, RB1, ERBB2, and FANCC, which are associated with radiotherapy, between the NCAPG groups (Figure 6(d)); ARID1A, ATM, ERBB2, RB1, ERCC2, and FANCC were more frequently mutated in the high NCAPG group. We compared the differences in EGFR_network, immune_inhibit_oncogenic_pathways, and radiotherapy_predicted_pathways between the groups (Figure 6(e)). The high NCAPG group correlated positively with DNA_replication and cell_cycle pathways among the radiotherapy_predicted_pathways type, while the trend was opposite in the low NCAPG group.

### 3.5. Identification of Immune-Related DEGs and PPI Network Analysis

**Figure 7:**
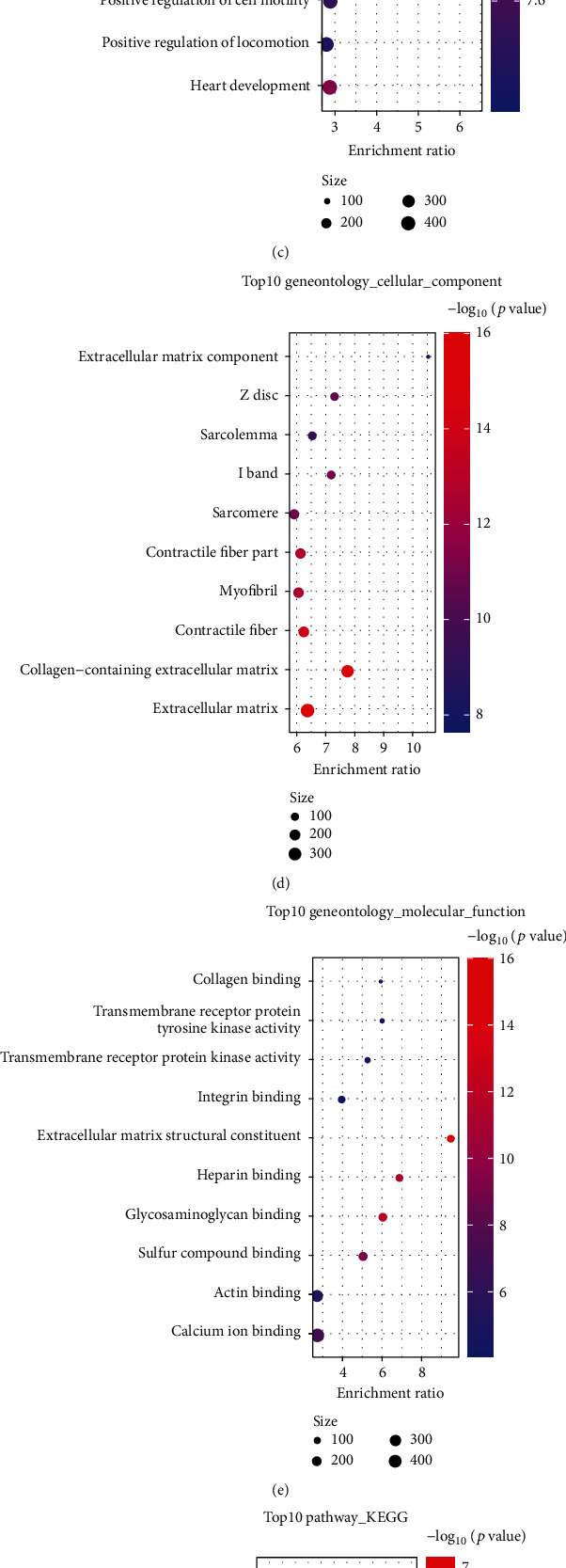
Immune-related differential gene expression analysis. (a) Intersection of upregulated genes among NCAPG, StromalScore, and ImmuneScore groups; (b) the intersection of downregulated genes among NCAPG, StromalScore, and ImmuneScore groups; (c–f) GO annotation and KEGG functional enrichment analyses for DEGsin NCAPG, StromalScore, and ImmuneScore groups.

The ESTIMATE algorithm was used to compute the immune and stromal scores, and two groups corresponding to each of them were classified. In the correlation analysis, expectably, we observed a significantly negative correlation of NCAPG expression with immune score and stromal score (*R* = −0.178 and -0.352, respectively, Fig. S2). We next divided STAD samples into two groups according to the cutoff values determined by survminer R package of NCAPG expression, immune score, and stromal score, respectively. Differential expression analysis identified the upregulated and downregulated DEGs between low and high NCAPG expression groups, low- and high-immune score groups, and low- and high-stromal score groups. Given that a negative correlation of NCAPG expression with immune score and stromal score was observed, we therefore performed an intersection analysis among upregulated DEGs of NCAPG group and downregulated DEGs of immune and stromal groups. A total of 19 DEGs were overlapped in the above three groups (Figure 7(a)). By using the same analysis, we identified 430 overlapping in downregulated DEGs of NCAPG group and upregulated DEGs of immune and stromal groups (Figure 7(b)).

**Figure 8:**
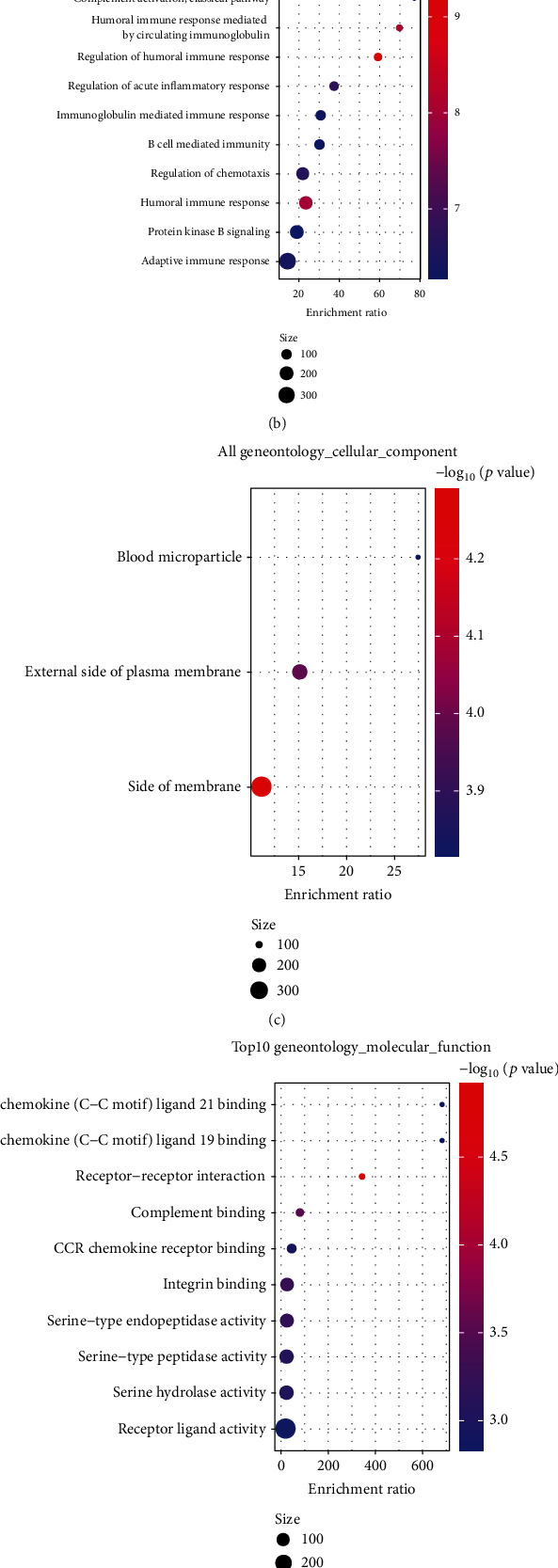
Module and functional analyses. (a) PPI network analysis graph for module Mcode3; (b–e) GO and KEGG functional enrichment analyses for genes in module Mcode3.

GO annotation and KEGG functional enrichment analyses for the DEGs were performed using WebGestaltR. Genes were closely related to pathways of tumorigenesis and immune responses (Figures 7(c)–7(f)), including regulation of angiogenesis, B cell receptor signaling pathway, microRNAs in cancer, and pathways in cancer. STRING was used for PPI analysis of 449 obtained DEGs. Cytoscape was utilized for network visualization and the MCODE plugin for identifying the key clusters. Five clusters with more than 10 genes (Mcode1, 2, 3, 5, and 9) were identified. KEGG functional enrichment analysis using WebGestaltR was performed to identify their functions. The Mcode3 module was closely related to immune and tumor pathways, including breast cancer, proteoglycans in cancer, and chemokine signaling (Figure 8).

### 3.6. IRS Model for STAD

**Figure 9:**
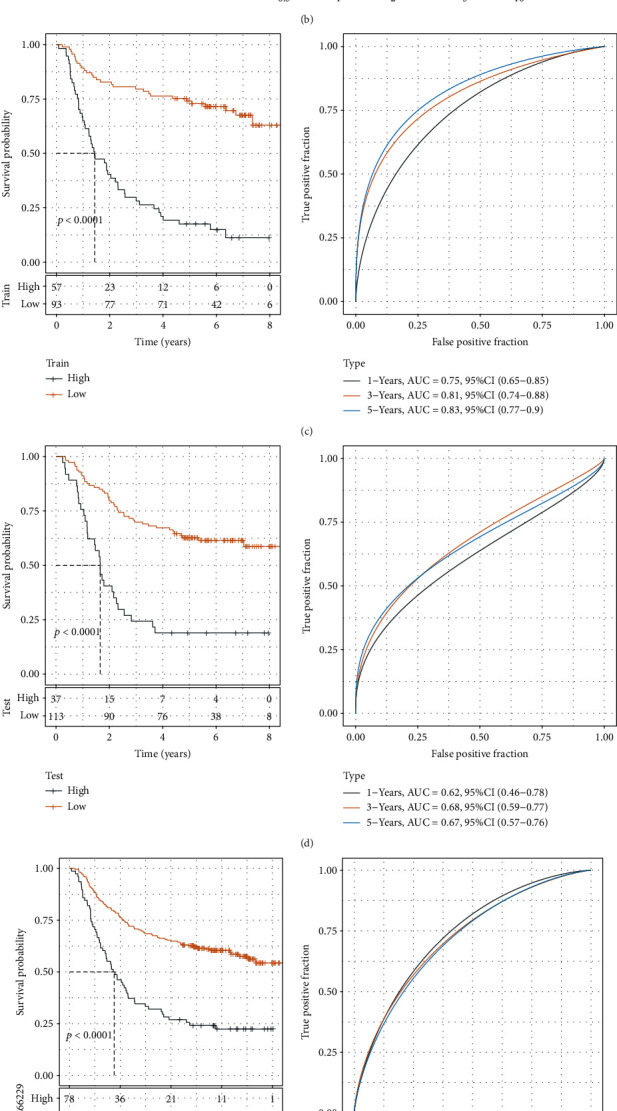
Construction of IRS for STAD. (a) Distribution of LASSO coefficients for 40 prognostic RNAs in the GEO training cohort. Coefficient profiles were plotted based on log(*λ*) sequences. (b) Multifactorial results of genes for the final IRS model. (c) KM and ROC analyses for the IRS model using the GEO training dataset. (d) KM and ROC analyses for the IRS model using the GEO validation dataset. (e) KM and ROC analyses for the IRS model using the entire GEO dataset. (f) KM and ROC analyses for the IRS model using the entire TCGA cohort.

After the above analysis, 449 immune-related differential genes were obtained, and univariate analysis revealed 248 prognosis-related genes (*P* < 0.05). The top genes were further selected using LASSO, and 10 genes were obtained according to the minimum lambda = 0.07841627 (Figure 9(a)). These were subjected to multifactorial analysis, and their risk coefficients were obtained (Figure 9(b)). IRS risk models were constructed according to the expression of these 10 genes. We calculated the risk score for each sample in the training and validation datasets of GSE66229 and divided the samples into high and low-risk groups according to the best cutoff. KM and ROC curves were plotted, wherein the AUC of one-year survival was 0.75, 0.81 for three years, and 0.83 for five years in the training set, while the corresponding values of one, three, and five years were 0.62, 0.68, and 0.67 (Figures 9(c) and 9(d)) in the validation set. Both in training and validation sets, the survival rate was higher for the low-risk group (*P* < 0.0001). The IRS model was validated using the entire GSE66229 and TCGA cohorts (Figures 9(e) and 9(f)). The STAD tumor samples could be clearly classified into two risk groups with different prognoses (*P* < 0.0001); the low-risk group showed a higher survival rate. For GSE84437, GSE26942, GSE13861, GSE28541, and GSE26253 datasets, the IRS scores for each sample were recalculated and KM and ROC curves were plotted. The corresponding AUCs for one-year survival were 0.64, 0.63, 0.66, 0.71, and 0.65; 0.65, 0.65, 0.8, 0.82, and 0.68 for three years, and 0.65, 0.66, 0.76, 0.89, and 0.69, for five years, respectively (Fig. S3).

### 3.7. The Relation between Risk Score and Tumor Stemness

**Figure 10:**
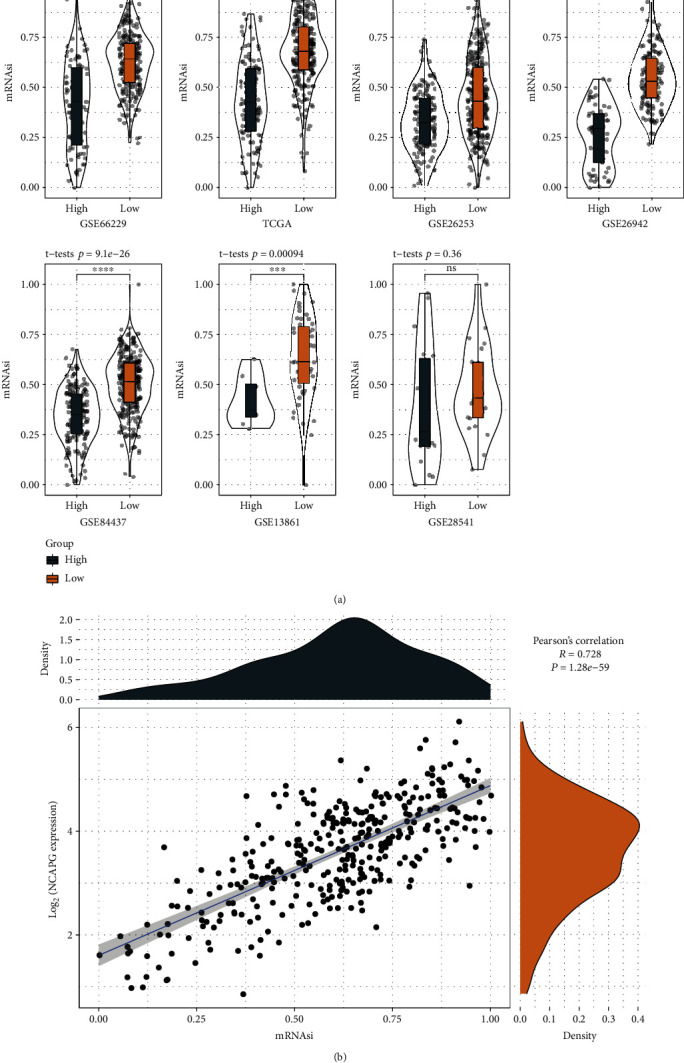
The relation between risk score and tumor stemness. (a) Comparison of tumor stemness (mRNAsi) between high- and low-risk groups in GSE66229, TCGA, GSE84437, GSE26942, GSE13861, GSE28541, and GSE26253 datasets. Student's *t*-test was conducted. (b) Pearson correlation analysis between NCAPG expression and mRNAsi. ns: not significant. ^∗∗∗^*P* < 0.001 and ^∗∗∗∗^*P* < 0.0001.

Tumor stemness has been revealed to be correlated with prognosis, which is linked with tumor microenvironment and oncogenic pathways such as Wnt/*β*-catenin and EMT pathways [23]. We calculated the mRNAsi scores of STAD samples in different datasets and compared their distribution in high- and low-risk groups. The results showed that the low-risk group had significantly higher mRNAsi scores than the high-risk group in all independent datasets including GSE66229, TCGA, GSE84437, GSE26942, GSE13861, GSE28541, and GSE26253 datasets (Figure 10(a)). In addition, we observed that NCAPG expression was positively correlated with mRNAsi (*R* = 0.728, *P* < 0.0001, Figure 10(b)). The results suggested that high degree of tumor stemness was associated with favorable prognosis in STAD.

### 3.8. Comparative Performances of IRS and TIDE

**Figure 11:**
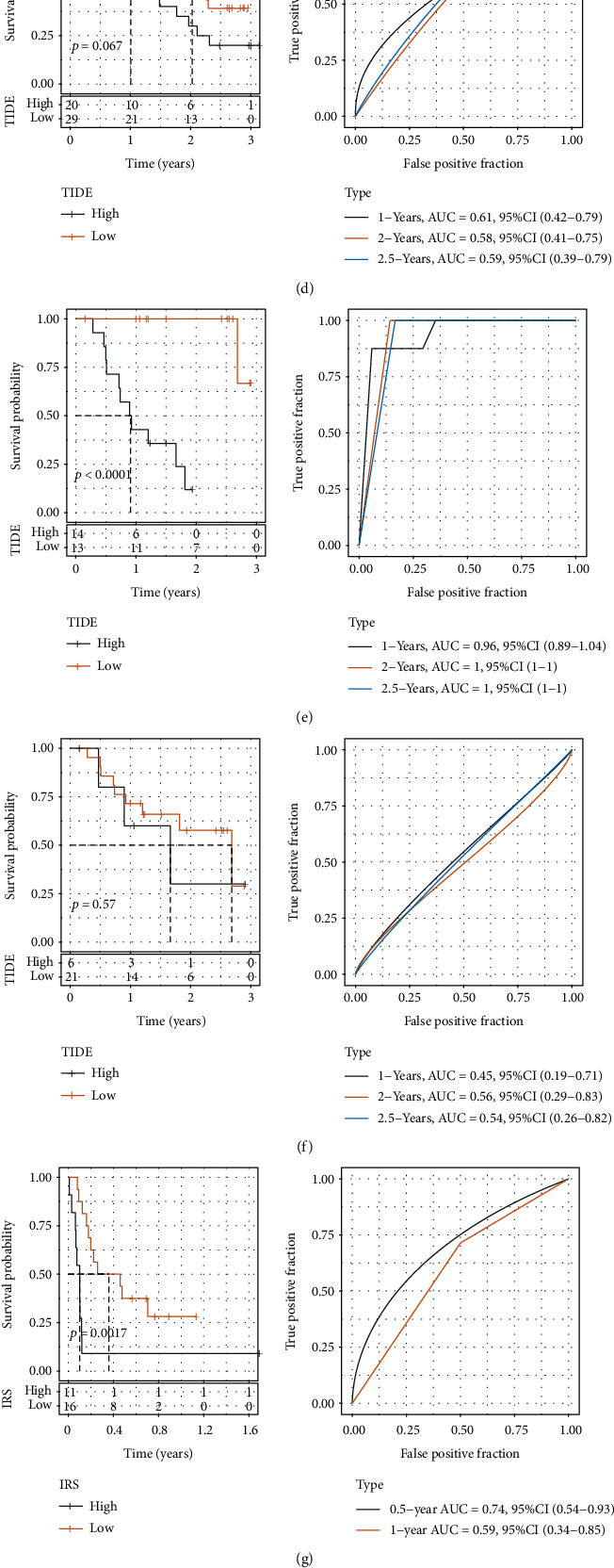
Comparative analysis of IRS and TIDE. (a) IRS survival and ROC curves for the IMvigor210 dataset. (b) TIDE survival and ROC curves for the IMvigor210 dataset. (c) IRS survival and ROC curves for the GSE91061 dataset. (d) TIDE survival and ROC curves for the GSE91061 dataset. (e) IRS survival and ROC curves for the GSE78220 dataset. (f) TIDE survival and ROC curve in the GSE78220 dataset. (g) IRS survival and ROC curves for the GSE135222 dataset. (h) TIDE survival and ROC curves for the GSE135222 dataset. Comparison of IRS and TIDE ROC curves in (i) IMvigor210, (j) GSE91061, (k) GSE78220, and (l) GSE135222 datasets.

To predict, evaluate, and compare efficacy scores for immunotherapy, the IMvigor210, GSE91061, GSE78220, and GSE135222 cohorts were selected as these comprised patients who underwent immunotherapy. We calculated IRS and TIDE score for each tumor sample in these cohorts and compared the efficiency of IRS and TIDE in the predicting the prognosis of the patients receiving immunotherapy. The samples were categorized into high- and low-scoring groups according to the best cutoff of IRS and TIDE scores. As a result, IRS was robust to distinguish high- and low-risk patients while TIDE score was only effective to predict the prognosis in the IMvigor210 dataset (Figures 11(a)–11(h)). The comparison of AUC values also suggested IRS had a superior performance than TIDE score in classifying the patients treated by immunotherapy into different risk groups (Figures 11(i)–11(l)). Therefore, IRS had a potential to predict the prognosis for not only untreated patients but immunotherapy-treated patients in STAD.

## 4. Discussion

In 21 cancer types, especially, STAD, NCAPG expression was significantly higher in tumor samples relative to the para-cancer samples; it was positively correlated with all four types of immune-related genes. Therefore, we used STAD as the case study. STAD is one of the most prevalent gastrointestinal malignancies, ranking fifth among incidences and third in mortality worldwide [24, 25]. Although 5-year survival rates are 90-97% when diagnosed and treated early, nearly 30% of patients with STAD are diagnosed at the advanced and metastatic stages [24, 25]. The malignant feature of STAD is determined not only by the activation and recruitment of immune and stromal cells, the major components necessary for tumor development and progression, in the tumor-associated microenvironment but also by the intrinsic activity of cancer cells [26]. Growing evidence confirms that the tumor microenvironment exerts an important effect on the development, progression, prognosis, and immune therapeutic responses in STAD [27, 28]. Stromal cell functions and interactions with tumor cells contribute to the progression, invasion, and spread of the tumor cells [29]. Stromal cells can secrete growth factors, cytokines, and chemokines which have a significant impact on tumor characteristics [30]. In STAD, NCAPG was significantly positively correlated with some chemokines. Therefore, NCAPG is a potentially new and reliable biomarker to predict the development and prognosis of STAD.

Several recent studies have focused on tumor immunology, and many immune checkpoint inhibitors have been developed which show robust and durable responses in patients with different cancers [31, 32]. This is consistent with the results reported herein and corroborates the reliability of our findings. In STAD, immune checkpoint genes including CD80, CTLA4, IDO1, and CD274 correlated positively with NCAPG expression. Based on clinical trials for immune checkpoint inhibitors for various cancers, immune cell infiltration in the tumor microenvironment is now considered valuable information for predicting prognosis and responses to immunotherapy [33, 34]. Therefore, we performed a comprehensive analysis of the overall immune cell infiltration in the tumor microenvironment by estimating the distributional differences of 28 immune cell scores in STAD among NCAPG subgroups. Twenty-one immune cell types showed significant differences between NCAPG grouping; the NCAPG low expression group had high immune scores. Macrophages act as immunosuppressive cells and they impede the activation of NK and CD8+ T cells; most of their marker genes were highly expressed in the NCAPG low expression group [35]. Immunosuppressive cells can respond to changes in immune cell abundances and occupy a central position in the tumor immune microenvironment. Therefore, we hypothesized that the poor prognosis in low NCAPG expression group may be related to this tumor immunosuppressive microenvironment.

Cancer stem cells (CSCs) compose only a fraction of 0.01-2% of tumor tissues in most tumors, but have great potential in initiating tumor growth [23]. It has been shown that tumor stemness varies in different cancer types [36]. In STAD, mRNAsi is suggested as a protective factor that high mRNAsi is associated with good prognosis [36, 37]. In our study, we found a significantly positive correlation of NCAPG expression with mRNAsi and high NCAPG expression group had a good prognosis, which was consistent with the previous findings. In high- and low-risk groups divided by the immune risk model, mRNAsi was higher in the low-risk group than in the high-risk group, which further supported mRNAsi as a protective factor in STAD. At the same time, the results also revealed the important role of NCAPG in tumor stemness. Experimental work has illustrated that quiescent tissue SCs escape from immune elimination resulting from systemic downregulation of MHC class I and TAP proteins that are responsible for antigen presentation [38]. Our results showed that the low NCAPG expression group had lower expression of some MHC class I genes such as HLA-A, HLA-B, HLA-C, HLA-F, HLA-G, TAP1, and TAP2 than the high NCAPG expression group, which may explain the worse prognosis of low NCAPG expression group. Our findings proposed a link of NCAPG expression with tumor stemness.

Although we have integrated and investigated data from different databases, certain limitations remain in our work. First, although bioinformatics provided useful insights into NCAPG expression in cancer, in vivo and in vitro biological experiments are required to validate our results to facilitate clinical applications. Mechanistic studies are warranted to elucidate the function of NCAPG at cellular and molecular levels. Specifically, the lack of support from prospective clinical studies may be compensated if actual patient data are available. In the future, emphasis will be on the role of NCAPG in STAD progression and regulation of the tumor microenvironment through ex vivo experiments.

In conclusion, we validated that NCAPG is differentially expressed between normal and tumor tissues; the results suggested the correlation between its expression and clinical prognosis in STAD. Our results suggested that the high level of NCAPG expression leads to different prognostic outcomes. With the help of the IRS model and the TIDE algorithm, NCAPG was shown to be effective in predicting immunotherapeutic responses in the STAD cohort. Together, the findings suggest that NCAPG is a valid biomarker for predicting immunotherapeutic responses. These results have implications for elucidating the role of NCAPG in tumorigenesis and progression especially in STAD and provide a reference for achieving precise and personalized immunotherapy in the future.

## 5. Conclusion

In conclusion, we explored a close relation of NCAPG with tumor microenvironment and prognosis as well as tumor stemness in STAD. We performed a comprehensive evaluation of the pan-cancer potential of NCAPG as a predictive biomarker and identified its significant value in STAD, which may have implications for immunotherapy and is expected to provide a useful assessment system for clinical application.

## Data Availability

The datasets generated and/or analyzed during the current study are available in the GSE66229 repository (https://www.ncbi.nlm.nih.gov/geo/query/acc.cgi?acc=GSE66229), in the GSE84437repository (https://www.ncbi.nlm.nih.gov/geo/query/acc.cgi?acc=GSE84437), in the GSE26942 repository (https://www.ncbi.nlm.nih.gov/geo/query/acc.cgi?acc=GSE26942), in the GSE13861 repository (https://www.ncbi.nlm.nih.gov/geo/query/acc.cgi?acc=GSE13861), in the GSE28541 repository (https://www.ncbi.nlm.nih.gov/geo/query/acc.cgi?acc=GSE28541), in the GSE26253 repository (https://www.ncbi.nlm.nih.gov/geo/query/acc.cgi?acc=GSE26253), in the GSE135222 repository (https://www.ncbi.nlm.nih.gov/geo/query/acc.cgi?acc=GSE135222), in the GSE91061 repository (https://www.ncbi.nlm.nih.gov/geo/query/acc.cgi?acc=GSE91061), in the GSE78220 repository (https://www.ncbi.nlm.nih.gov/geo/query/acc.cgi?acc=GSE78220), and in the GSE84437 repository (https://www.ncbi.nlm.nih.gov/geo/query/acc.cgi?acc=GSE84437).

## Supplementary Materials

Supplementary 1 FigS1: Kaplan-Meier survival curves of high and low NCAPG expression groups in 33 cancer types.

Supplementary 2 FigS2: Pearson correlation analysis of NCAPG expression with immune score and stromal score.

Supplementary 3 FigS3: the performance of IRS model in GSE84437, GSE26942, GSE13861, GSE28541, and GSE26253 datasets.
