# Supplementary figures and images for: Characterizing the Crosstalk of NCAPG with Tumor Microenvironment and Tumor Stemness in Stomach Adenocarcinoma
Source: Stem Cells Int. 2022 Oct 3;2022:1888358. doi: 10.1155/2022/1888358 (PMC9551677; doi:10.1155/2022/1888358)

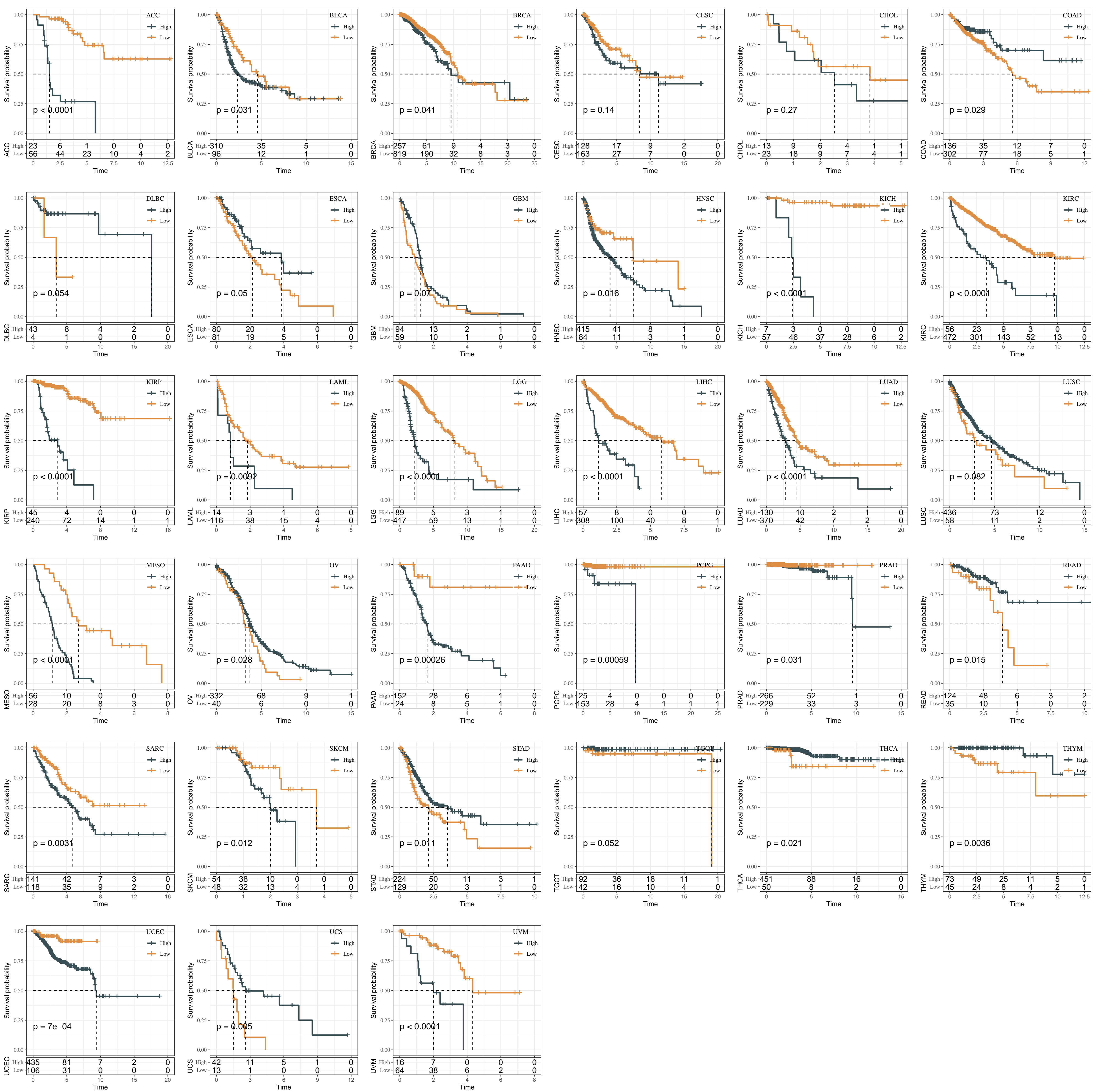

Supplement: Supplementary 1 — FigS1: Kaplan-Meier survival curves of high and low NCAPG expression groups in 33 cancer types. [file 1888358.f1.pdf]

**A**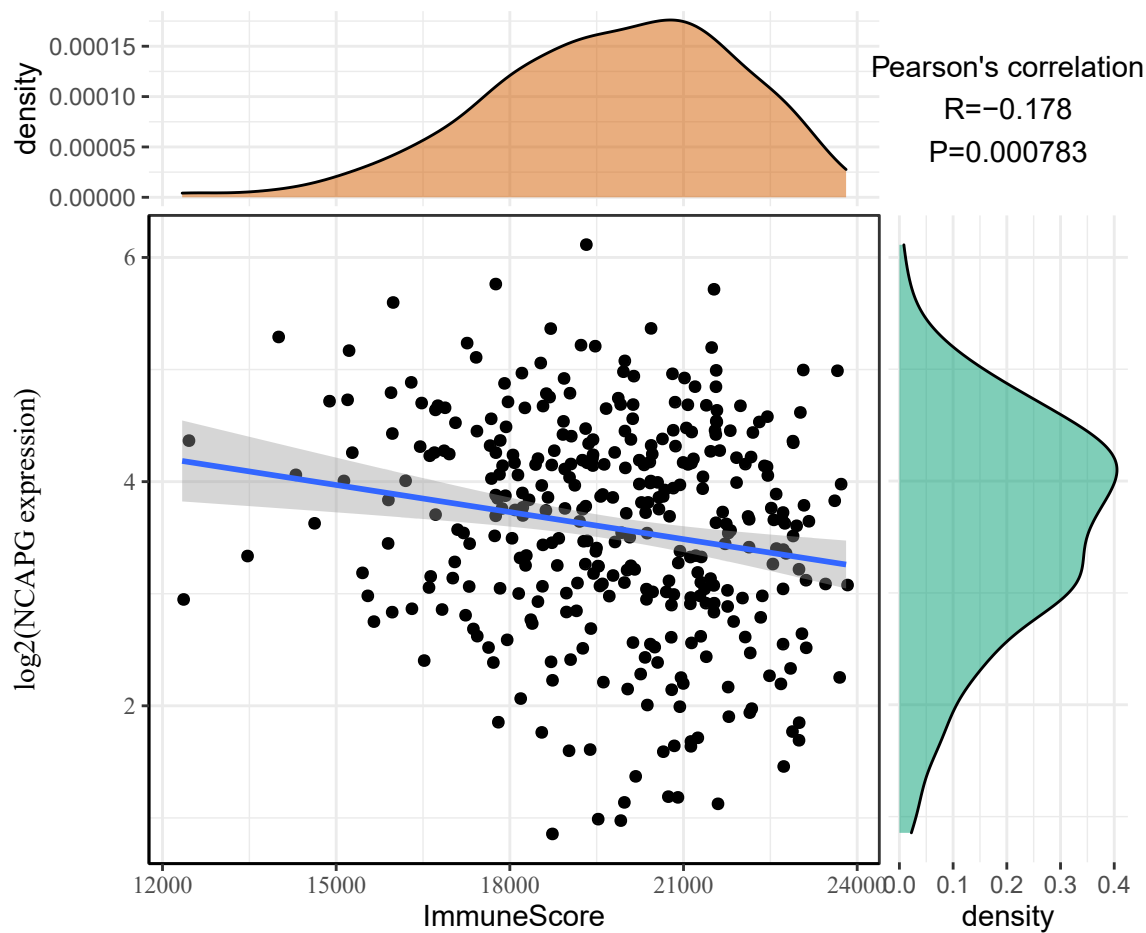**B**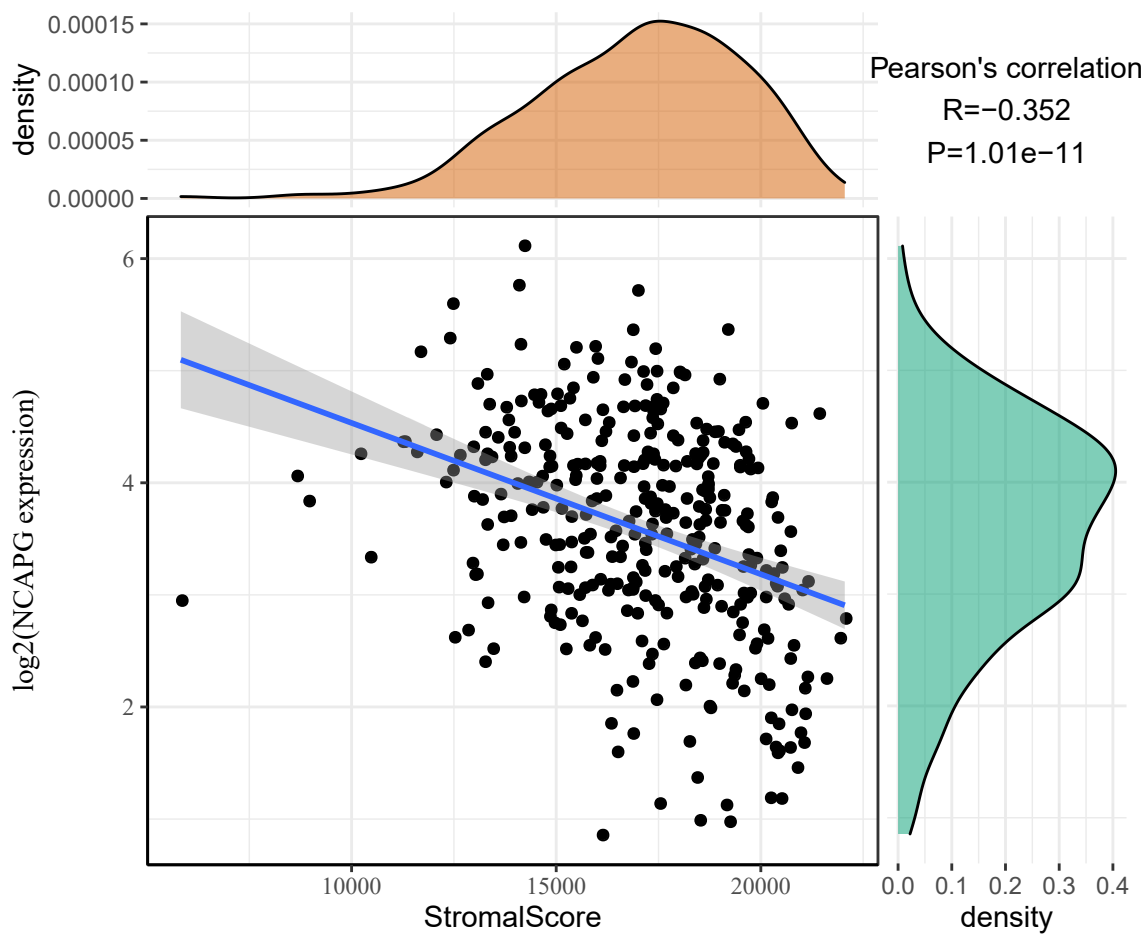

Supplement: Supplementary 2 — FigS2: Pearson correlation analysis of NCAPG expression with immune score and stromal score. [file 1888358.f2.pdf]

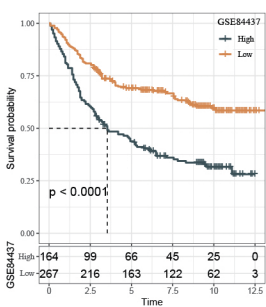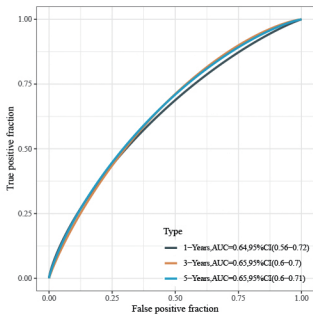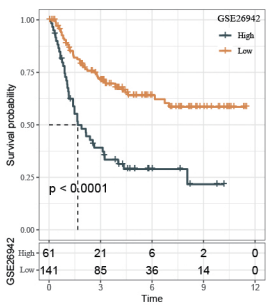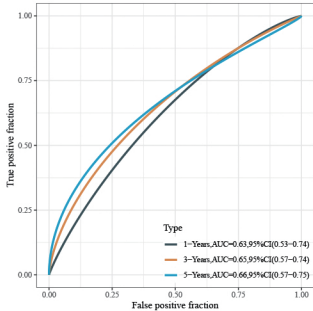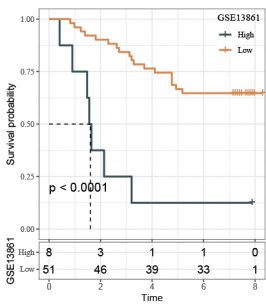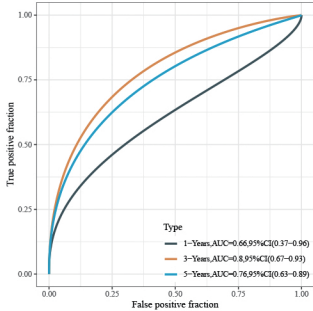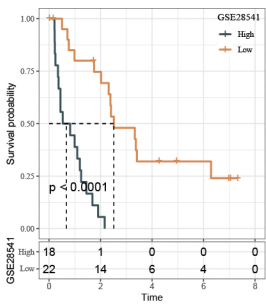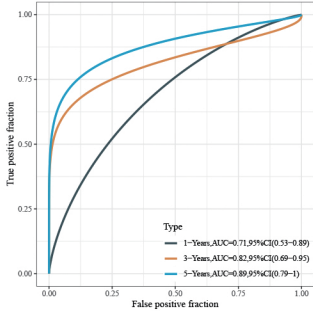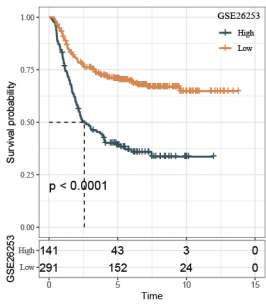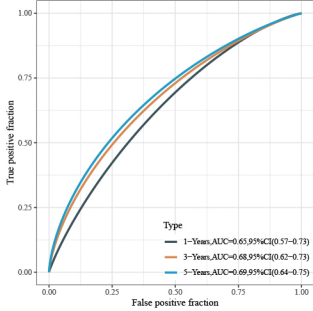

Supplement: Supplementary 3 — FigS3: the performance of IRS model in GSE84437, GSE26942, GSE13861, GSE28541, and GSE26253 datasets. [file 1888358.f3.pdf]
